# Minnelide/Triptolide Impairs Mitochondrial Function by Regulating SIRT3 in P53-Dependent Manner in Non-Small Cell Lung Cancer

**DOI:** 10.1371/journal.pone.0160783

**Published:** 2016-08-08

**Authors:** Ajay Kumar, Catherine Corey, Iain Scott, Sruti Shiva, Jonathan D’Cunha

**Affiliations:** 1 Department of Cardiothoracic Surgery, University of Pittsburgh, Pittsburgh, Pennsylvania, United States of America; 2 Vascular Medicine Institute, University of Pittsburgh, Pittsburgh, Pennsylvania, United States of America; 3 Dept of Pharmacology & Chemical Biology, University of Pittsburgh, Pittsburgh, Pennsylvania, United States of America; Roswell Park Cancer Institute, UNITED STATES

## Abstract

Minnelide/Triptolide (TL) has recently emerged as a potent anticancer drug in non-small cell lung cancer (NSCLC). However, the precise mechanism of its action remains ambiguous. In this study, we elucidated the molecular basis for TL-induced cell death in context to p53 status. Cell death was attributed to dysfunction of mitochondrial bioenergetics in p53-deficient cells, which was characterized by decreased mitochondrial respiration, steady-state ATP level and membrane potential, but augmented reactive oxygen species (ROS). Increased ROS production resulted in oxidative stress in TL-treated cells. This was exhibited by elevated nuclear levels of a redox-sensitive transcriptional factor, NF-E2-related factor-2 (NRF2), along with diminished cellular glutathione (GSH) content. We further demonstrated that in the absence of p53, TL blunted the expression of mitochondrial SIRT3 triggering increased acetylation of NDUAF9 and succinate dehydrogenase, components of complexes I and II of the electron transport chain (ETC). TL-mediated hyperacetylation of complexes I and II proteins and these complexes displayed decreased enzymatic activities. We also provide the evidence that P53 regulate steady-state level of SIRT3 through Proteasome-Pathway. Finally, forced overexpression of Sirt3, but not deacetylase-deficient mutant of Sirt3 (H243Y), restored the deleterious effect of TL on p53-deficient cells by rescuing mitochondrial bioenergetics. On contrary, Sirt3 deficiency in the background of wild-type p53 triggered TL-induced mitochondrial impairment that echoed TL effect in p53-deficeint cells. These findings illustrate a novel mechanism by which TL exerts its potent effects on mitochondrial function and ultimately the viability of NSCLC tumor.

## Introduction

Minnelide/Triptolide (TL), a diterpenoid triepoxide, was first extracted from a traditional Chinese Médicinal plant Tripterygium wilfordii Hook For Thunder God Vine [1]. It has been well documented that TL possesses a broad-spectrum therapeutic potential because of its anti-inflammatory, immunosuppressive, and anti-tumor activities [2]. Therefore, its cytotoxic effect has been demonstrated in a wide variety of epithelial and hematological malignancies, including pancreatic [3, 4], gastric [5], colorectal cancer cells [6], as well as in neuroblastoma [7, 8], and NSCLC [9, 10]. In addition, TL has been shown to be the most potent inhibitor of lung inflammation in acute lung injury models [11–13]. TL achieves these beneficial properties by regulating multiple key proteins. For example, TL inhibits heat shock proteins, survivin, AKT, c-myc and pRB [14–17]. Because TL is only soluble in organic solvent, a water-soluble derivative has been developed called Minnelide [18]. Recently, we have provided evidence that Minnelide/TL significantly reduced the expression of pro-survival and anti-apoptotic genes, whereas up-regulated pro-apoptotic genes in non-small cell lung carcinoma (NSCLC) [10] via mitigating the NF-kB signaling. Despite considerable advances in research for TL in the field of cancer, the precise mechanism of how TL modulates cytotoxicity in NSCLC is still incompletely defined.

Mitochondria generate cellular energy in the form of ATP utilizing substrates from tricarboxylic acid (TCA) which drive oxidative phosphorylation (OXPHOS) [19]. OXPHOS is catalyzed by the electron transport chain, which consists of five mitochondrial protein complexes (I-V) and is the major ATP producer under physiologic conditions. While complexes I-IV expedite the reduction of oxygen and the translocation of H^+^ from the matrix to the intermembrane space to generate a proton gradient, complex V (F_1_F_0_-ATP-synthase) utilizes these protons to synthesize ATP [20]. In addition to ATP production, mitochondria also mediate cell death and produce reactive oxygen species (ROS), which can be harmful for the cells if produced excessively [21]. In cancer cells, rapidly growing malignant cells are thought to constitutively switch from OXPHOS to glycolysis. In recent years, substantial efforts have been directed towards new anticancer drugs that target OXPHOS and glycolysis in rapidly growing cancer cells. In this direction, two approaches have been proposed. The first approach is to activate OXPHOS leading to accumulation of ROS and subsequent death [22]. In the second approach, drug treatment of cancer cells decreases both glycolysis and OXPHOS to induce an overall energy deficiency leading to death [23].

In mitochondria, several ETC components are modified by post-translational modifications (PTMs). Such modifications of mitochondrial proteins regulate their activities, stability and subcellular localization [24]. Among these modifications, reversible acetylation of mitochondrial protein is emerging as a major PTM and is regulated by acetyltransferases and deacetylases [25, 26]. Recently, the class III histone deacetylases, the Sirtuins, have appeared as major deacetylases [27, 28]. There are seven mammalian sirtuins: Sirt 1,6 and 7 are localized to the nucleus; Sirt 2 is mainly in cytoplasm; whereas Sirt 3,4 and 5 are prominently in mitochondria [28]. Given the fact that Sirt3-deficient mice (but not Sirt4 or 5) display extensive hyperacetylation of mitochondrial proteins, Sirt3 seems to be the major mitochondrial deacetylase [29–31]. Sirt3 regulates mitochondrial respiration and ATP production [32]. Mechanistically, Sirt3 controls the widespread acetylation of mitochondrial proteins including the subunits of electron transport chain (ETC) that leads to altered activities of these complexes [32–34]. Recent studies have emphasized the ability of Sirt3 to protect cells from oxidative damage by regulating antioxidant proteins, superoxide dismutase 2 (SOD2), catalase and isocitrate dehydrogenase-2 (IDSH2) suggesting a crucial role of Sirt3 in regulating ROS homeostasis [8, 35]. Given the fact that mitochondrial ROS-mediated oxidative stress plays a major role in cancer, Sirt3 may indeed significantly impact tumor proliferation. Therefore, agents that modulate Sirt3 function may have some potential therapeutic benefits.

The p53 tumor suppressor gene is a transcription factor that is induced and activated in response to various cellular stresses. The functional inactivation of p53 is one of the most frequent molecular events of human cancer [36]. Therefore, unprecedented efforts have been devoted over the last many years to examine the signaling pathway(s) by which p53 exerts its anti-tumor effect. The growing interest in p53 is due to multiple reasons. For example, genetic inactivation of p53 is the leading cause of all malignancies [36]. A dysfunctional p53 pathway has been linked with increased resistance to chemo- and radiotherapy with increased genomic instability [37, 38]. In recent years multiple therapeutic approaches have been developed based on the fact that p53-depleted malignant cells demonstrated defective cell cycle regulation. For instance, cancer cells deficient in p53 are more prone to enter the S and M phase of cell cycle in response to DNA damaging agents. Therefore, various drugs have been proven to be more cytotoxic to p53-deficient cells as compared to their wild type counterparts [39]. P53 also plays a key role in mitochondrial energy metabolism by regulating metabolic events [40]. P53 can regulate OXPHOS through the modulation of complex activities. In addition, p53 has been documented to regulate glycolysis [40].

Intrigued by the possibility that certain drugs exert their cytotoxic effect in cancer cells by targeting mitochondrial function, we hypothesized that TL-mediated cellular toxicity in NSCLC is due to dysfunctional mitochondria and this effect was p53 dependent.

## Materials and Methods

### Cell culture and reagents

A549, NCI-H2009, NCI-H460, H1299 and BEAS-2B were obtained from ATCC. HCT116 cells (p53^+/+^ and p53^-/-^) were a gift from Dr. Bert Vogelstein (John Hopkins Institute, Baltimore, USA). Myc-tagged Sirt3 plasmids were kind gift from Dr. Toren Finkel (NHLBI, NIH, Bethesda, MD). P53 expression plasmids has been described before [41].Antibodies used were: β-actin, p53-DO1, NRF2, Citrate Synthase and HO-1 from Santa Cruz Biotechnology (Santa Cruz, CA); Anti-hNDFA9, Anti-SDHA, Anti-NQO1; Anti-MSH2 and anti-Cox IV from Abcam, USA; Anti-acetylated lysine and SIRT3 antibodies from Cell Signaling, USA; Anti-SOD2 antibody from Millipore, USA; Anti-SKP2 from Bethyl Laboratory, USA. Chemicals related to complexes assays, MG132, Cycloheximide and Mito-TEMPO were purchased from Sigma-Aldrich, US. Stock solution of MG132 and mito-TEMPO were prepared in DMSO (20 mM), whereas for cycloheximde 50 μg/ml solution was prepared in DMSO.

Triptolide (TL) was purchased from Calbiochem, NJ, USA and was dissolved in Dimethyl sulfoxide (DMSO from Sigma-Aldrich, St. Louis, MO, USA) to a stock solution of 1 mg/ml. Minnelide was a gift of Dr. Ashok Saluja (University of Minnesota, Minneapolis, MN). Cells were stimulated with indicated doses of TL in complete media. DMSO alone was also included to serve as a control.

### Cell Proliferation Assay

Cell viability was determined by using CyQUANT cell proliferation assay kit (Life Technologies, USA) according to manufacturer protocol.

### Seahorse XF-24 Metabolic Flux Analysis

Cells were treated with indicated amounts of TL for 4 hours and were subjected to measurement for oxygen consumption rate using an XF Analyzer (Seahorse Biosciences, USA). Non-ATP linked, maximal and non-mitochondrial respiration of the cells were measured by sequentially adding Oligomycin (2 μM), Carbonyl cyanide-p-trifluromethoxyphenylhydrazone (FCCP 0.25 μM) and Antimycin A + Rotenone (2.5 μM) respectively. In some experiment, cells were first transiently transfected with indicated plasmids or siRNA before stimulating with TL and then subjected for OCR analysis.

### ATP measurement

ATP was measured using an ENLITEN ATP assay system from Promega, USA. Briefly, cells were harvested in ice-cold ATP buffer [20 mM Tris (pH 7.5), 25 mM NaCl, 2.5 mM EDTA, 0.5% Nonidet P-40]. After being incubated on ice for 10 minutes, samples were centrifuged at 13,000 g for 15 minutes. Lysates were analyzed for ATP content using luminometer, normalized with protein amount and were presented as fold change over control vehicle-treated cells.

### Mitochondrial Membrane Potential (ΔΨ)

Following stimulation with TL, cells were processed to measure mitochondrial membrane potential using the JC-1 mitochondrial membrane potential assay kit (Cayman, USA) according to manufacturer’s protocol. Briefly, cells were labeled with JC1 dye at 37oC incubator for 15 minutes. JC1 fluorescence was measured first for J-aggregates with excitation/emission at 535nm/595 and then for J-monomer with excitation/emission at 485nm/535. The data was presented as ratio J-aggregates to J-monomer which has been used as an indicator of cell health

### Mitochondrial ETC Enzyme Complex Activities

Unstimulated and TL treated cells were harvested in STE buffer (250 mM sucrose, 10 mM Tris, 1 mM EGTA, pH 7.4) at 4°C. The enzyme activities of complexes I, II, IV and citrate synthase were measured by spectrophotometric kinetic assay as described before [42, 43].

### Measurement of Mitochondrial Reactive Oxygen Species (mtROS)

mtROS production was measured in control and treated cells using MitoSOX Red Mitochondrial superoxide indicator (Invitrogen, Carlsbad, CA; 5 μM) according to the manufacturer’s recommendation. Briefly, cells were washed in 1XHBSS (with Calcium and Magnesium) and labeled with MitoSOX. The fluorescent intensity (510/580 nm) was measured kinetically as described before and was normalized to the amount of protein used in the assay [42].

### Measurement of Glutathione levels

Glutathione (GSH) levels were measured in cell lysates from control or TL-stimulated cells using Glutathione Assay Kit from Cayman Chemical (USA) according to manufacturer’s recommendation. Briefly, cells were washed in cold PBS and were lysed using 1XGSH MES buffer on ice for 10 minutes. Cell lysates were centrifuged at 10,000xg for 15 minutes at 4°C and the supernatant was deproteinated using equal amount of 5% Metaphosphoric acid. After centrifugation, the resulting supernatant was neutralized with TEAM reagent (50 μl/ml) for the measurement of total glutathione. The GSH content was normalized to amount of protein and expressed as relative levels over DMSO treated cells.

### Measurement of Superoxide dismutase (SOD) activity

The SOD activity was measured in cell lysates from control or TL-stimulated cells using Superoxide Dismuatse Assay Kit from Cayman Chemicals (USA) according to manufacturer’s protocol. Briefly, cells were harvested in STE buffer (250 mM sucrose, 10 mM Tris, 1 mM EGTA, pH 7.4) and cells were homogenized in cold. Cell lysates were centrifuged at 1500Xg for 10 minutes at 4 degree C and activity was measured in cell lysates. SOD activity was expressed in U/mg of protein.

### siRNA-mediated Knockdown of Sirt3

Knockdown p53 and Sirt3 was performed as previously described [41]. Cells were plated 1 day before transfection in antibiotic-free media. On the day of transfection, 20 nM of specific siRNA targeting specific gene of interest (Sirt3, p53) or nonspecific siRNA was incubated with Lipofectamine 2000 (Invitrogen, CA) at room temperature for 30 min and transfection mixture was added in cells in Opti-MEM media. Three hour later, the medium was replaced by complete media and cultured for an additional 48 h. The knocked-down genes were confirmed by immunoblotting. Stealth siRNA against human p53 and Sirt3 along with the negative control-pool were purchased from Life Sciences, USA. The sequences of siRNA are: 5’- CCUGCAGGAUGUAGCUGAG CUGAUU-3’, 5′-AAUCAGCUCAGCUACAUC CUGCAGG-3′.

### Transient Transfection Reporter Assays

Cells were plated at a density of 5×10^4^ per well in 24-well plates 1 day before transfection. Transient transfection was performed using Lipofectamine2000. A total 1 to 2 μg of plasmid DNA was used in transfections, and total DNA was kept constant. Cotransfected Renilla luciferase was used as an internal control. Cells were harvested 24 hours after transfection, firefly and Renilla luciferase activity was measured using a dual luciferase reporter kit (Promega, USA), and firefly values were normalized to Renilla values in each sample.

### Immunoblotting and Immunoprecipitation Analysis

Immunoblotting was performed as described previously [41, 44]. Briefly, 50 μg of protein lysates from untreated cells and cells exposed to Triptolide were boiled in SDS-PAGE gel loading buffer (Bio-Rad), subjected to SDS-PAGE, transferred to nitrocellulose filter, and probed with the specified primary antibody and the appropriate peroxidase-conjugated secondary antibody (Santa Cruz Biotech). Chemiluminescent signal was developed using Super Signal West Femto substrate (Pierce), blots imaged with a Gel Doc 2000 Chemi Doc system (BioRad). For immunoprecipitation studies, cells were lysed in IP buffer [50mM Tris (pH 7.4), 150 mM NaCl, 5% Glycerol, 1% Triton-X100) containing Complete EDTA-free protease inhibitor mixture (Roche). Cell lysates were incubated with indicated antibodies for overnight at 4°C. Next day, immune complex was captured using Dynabeads Protein G (Invitrogen, USA) by incubating for additional 45 minutes. Immune complexes were washes four times, eluted with SDS-PAGE gel loading buffer and were separated by SDS-PAGE as described above. For endogenous immunoprecipitation of P53 and sirt3, cells were cross-linked with formaldehyde (1%) for 15 minutes to stabilize the complex before harvesting cells in IP buffer.

### Real-Time RT-PCR

Total RNA from cultured cells was isolated using RNeasy Kit (Qiagen, CA). cDNA was synthesized using qScript^™^ cDNA SuperMix (Qunata Biosciences, USA). Real-time PCR was performed using a SYBY Green mix to monitor the amplification on ABI-7500 PCR machine. The following primers were used: Human Sirt3 (Forward-TGCGGCAAGACCTACACCAAGAGT; Reverse-AGCCGCAGCCGTCCCAGTT) and Human GAPDH (Forward-CCACATCGCTCAGACACCAT; Reverse- CCAGGCGCCCAATACG). The data was normalized with GAPDH.

### Statistical methods and analysis

All qualitative data are acquired and representative of at least three independent experiments. Results are expressed as mean ± SD or mean ± SEM. Statistical analysis was performed with SigmaStat. Student t-test was used to compare between two groups. Data in which more than two conditions were compared in a single experiment were tested using ANOVA. A p value of <0.05 was considered statistically significant.

## Results

### Maximal cytotoxic effect of TL on NSCLC cells depended upon p53 status-

**Fig 1:**
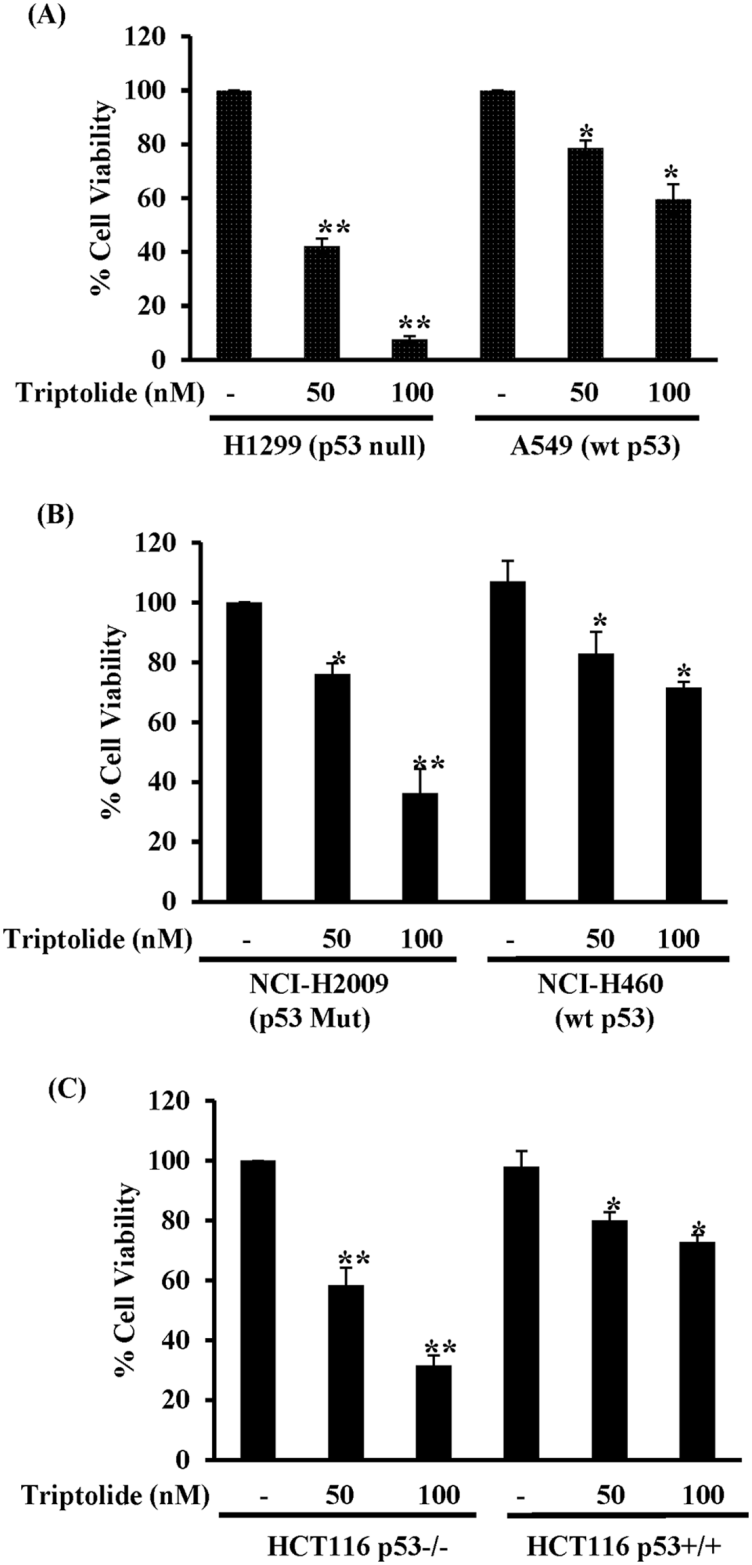
TL decreases cell viability in malignant cells dependent of p53 status. **(A-C)** A549, H1299, NCI-H460, NCI-H2009 and HCT116 (p53+/+ and p53-/-) cells were stimulated with TL (50 and 100 nM for 16 hours). Cell viability was measured by CyQUANT cell proliferation assay kit and data was presented as percent viable cells (mean ± SD) over control cells (DMSO treated). (*p<0.05, **p<0.001; n = 3).

Prior studies from our group have demonstrated that TL increased cell death in a dose- and time-dependent manner in multiple cancer cell lines including NSCLC [10, 45]. Because the tumor-suppressor p53 governs cell cycle arrest and cell viability in NSCLC, we asked whether p53 plays a part in TL-induced cell proliferation. For this purpose, we assessed the effect of TL treatment on cell viability of various malignant cell lines with different p53 status e.g. A549 (wt-p53), H1299 (p53 deficient cells), NCI-H460 (wt-p53), NCI-H2009 (mutant p53) of lung epithelial cancer cells and HCT116 with its isogenic p53 deficient cell line (HCT116 p53+/+ and HCT116 p53-/-) of colon epithelial cancer cell line. Strikingly, each of these cell lines displayed differential cellular toxicity based on p53 status. For example, as compared to wild type p53 lung epithelial cells (A549, NCI-H460), p53 null/mutant cells (H1299, NCI-H2009) demonstrated significantly decreased cell viability in the presence of similar doses of TL (Fig 1A and 1B). Very similar results were observed when HCT116 p53+/+ and its isogenic HCT116 p53-/- were stimulated with TL. Decreased cell viability was exhibited in TL-treated p53-/- cells as compared to p53+/+ cells (Fig 1C). Overall, these findings suggested that TL exerts its deleterious effect in p53-deficient/mutant transformed cells.

### TL promoted mitochondrial dysfunction selectively in p53-deficient cells

**Fig 2:**
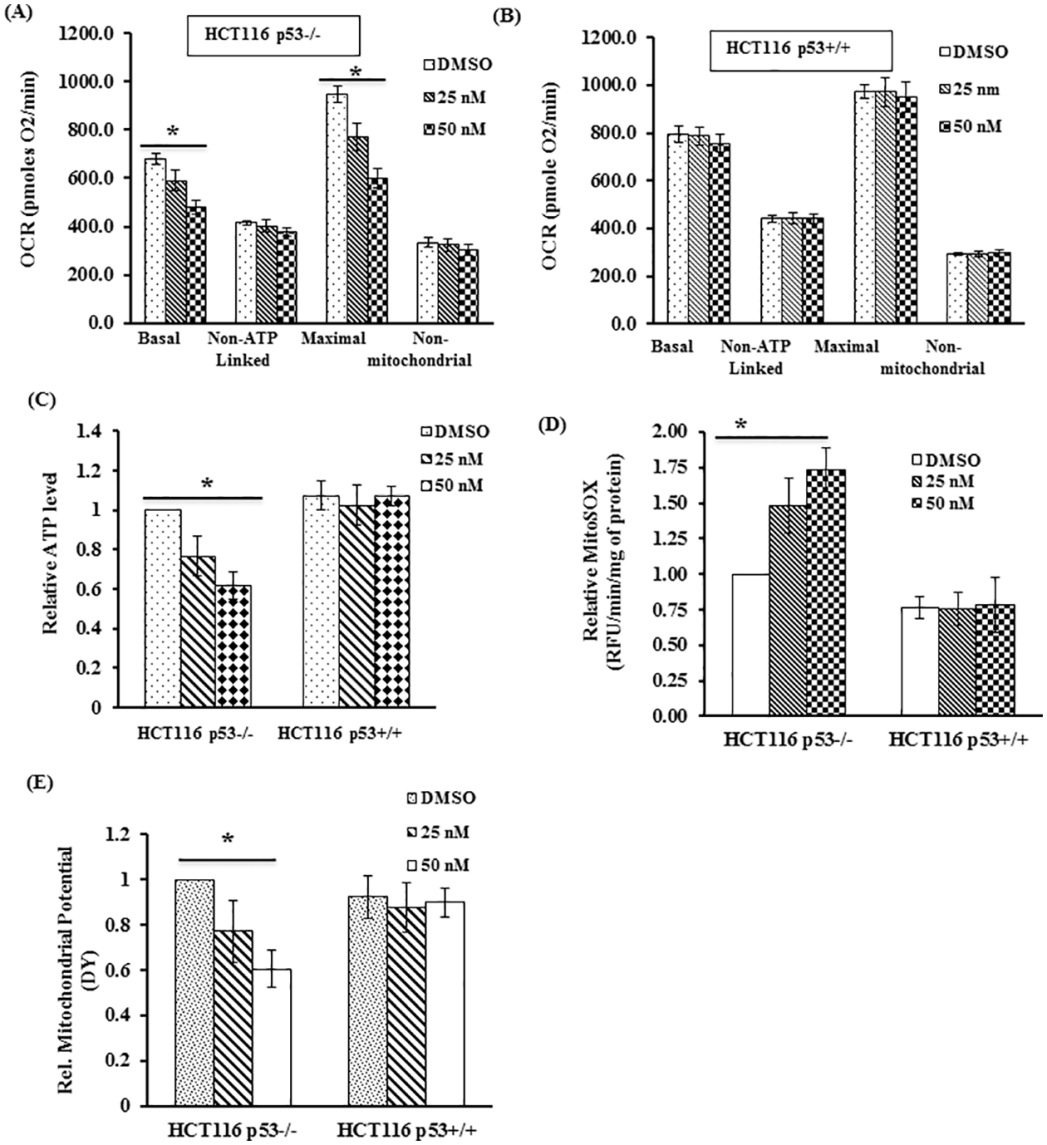
TL stimulation causes mitochondrial dysfunction in p53 deficient cells. (A) HCT116 p53-/- and (B) HCT116 p53+/+ treated with DMSO or indicated doses of TL for 6 hours were analyzed for oxygen consumption rate (OCR) using the Seahorse Bioscience Extra Cellular Flux analyzer. Data were presented as basal, non-ATP-linked, maximal and non-mitochondrial respiration (mean±SEM; *p<0.05; n = 3). (C) ATP levels and (D) Mitochondrial ROS (mtROS) were measured in TL-treated (6 hours) and untreated HCT116 cells using MitoSOX assay. Relative MitoSOX fluorescence values were presented as fold change over control. (E) Mitochondrial membrane potential (ΔΨ) were measured in HCT116 p53+/+ and p53-/- cells following treatment with or without TL (25, 50 nM for 6 hours). Values, normalized to protein content, were quantified and were expressed as fold change compared to control (untreated) cells (mean±SD; *p<0.05; n = 3).

In efforts to fully elucidate the role of p53 in TL-induced cell death, we focused on HCT116 p53+/+ and its isogenic p53-/- clone for the remainder of the study. To assess the possible impact of TL treatment on mitochondrial function, we first measured mitochondrial respiration by determining oxygen consumption rate (OCR) in control and TL stimulated HCT116 cells using Seahorse XF24 analysis. In these experiments, we chose to use lower doses of TL for 6 hours so as to be able to dissect the events more fully. P53 deficient HCT116 cells (p53-/-) displayed significantly lower basal and maximal respiration in the presence of TL when compared with DMSO treated cells (Fig 2A and S1 Fig). Intriguingly, OCR (basal and maximal) was unaltered in TL-treated HCT116 p53+/+ cells (Fig 2B and S1 Fig). Consistent with OCR data, steady state cellular ATP levels were significantly decreased in TL-treated HCT116p53-/- cells compared to unstimulated cells (Fig 2C). However, no such noticeable difference for ATP levels were observed between untreated and TL-treated p53+/+ cells (Fig 2C).

Since mitochondrial respiration is a significant source of mtROS through ETC, we determined whether decreased OCR in TL-induced p53-/- cells leads to changes in mtROS production. Indeed, as compared to unstimulated cells, significantly increased mtROS production was observed in TL treated p53-/- cells as confirmed by a MitoSOX kinetic assay (Fig 2D). Moreover, presence of p53 in wild type HCT 116 cells abolished the effect of TL on mtROS production (Fig 2D). To further dissect the significance of mtROS for noxious effect of TL, we explored the use of Mito-TEMPO, the mitochondrial-targeted antioxidant with superoxide scavenging properties. HCT116p53-/- cells were pretreated with Mito-TEMPO for 2 hours before stimulation with TL and were subjected for mitoSOX analysis and cell viability. Indeed, stimulation of p53 deficient cells with mito-TEMPO prior to TL treatment significantly reduces the mtROS production induced by TL (S2A Fig) and more importantly improves cell viability (S2B Fig). Once we confirmed decreased OCR and increased mtROS production mediated by TL, we next assessed mitochondrial membrane potential (ΔΨ). TL blunted the mitochondrial membrane potential of HCT116 p53-/- with no effect on HCT116 p53+/+ cells suggesting depolarization of the mitochondrial membrane by TL in HCT116 p53-/- cells (Fig 2E). Finally to assess effect of TL for mitochondrial function in NSCLC, H1299 cells were stimulated with TL (50 nM) for 6 hours and were assessed for OCR, mtROS, and ATP levels. Similar to HCT116 cells, TL stimulation exhibited impaired mitochondrial function with decreased OCR and ATP level, but increased mtROS (S3 Fig). Taken together, these results support that p53 protects the cells for TL-induced impaired mitochondrial bioenergetics.

### P53 mitigates TL-mediated mitochondrial dysfunction in p53 deficient HCT116 cells

**Fig 3:**
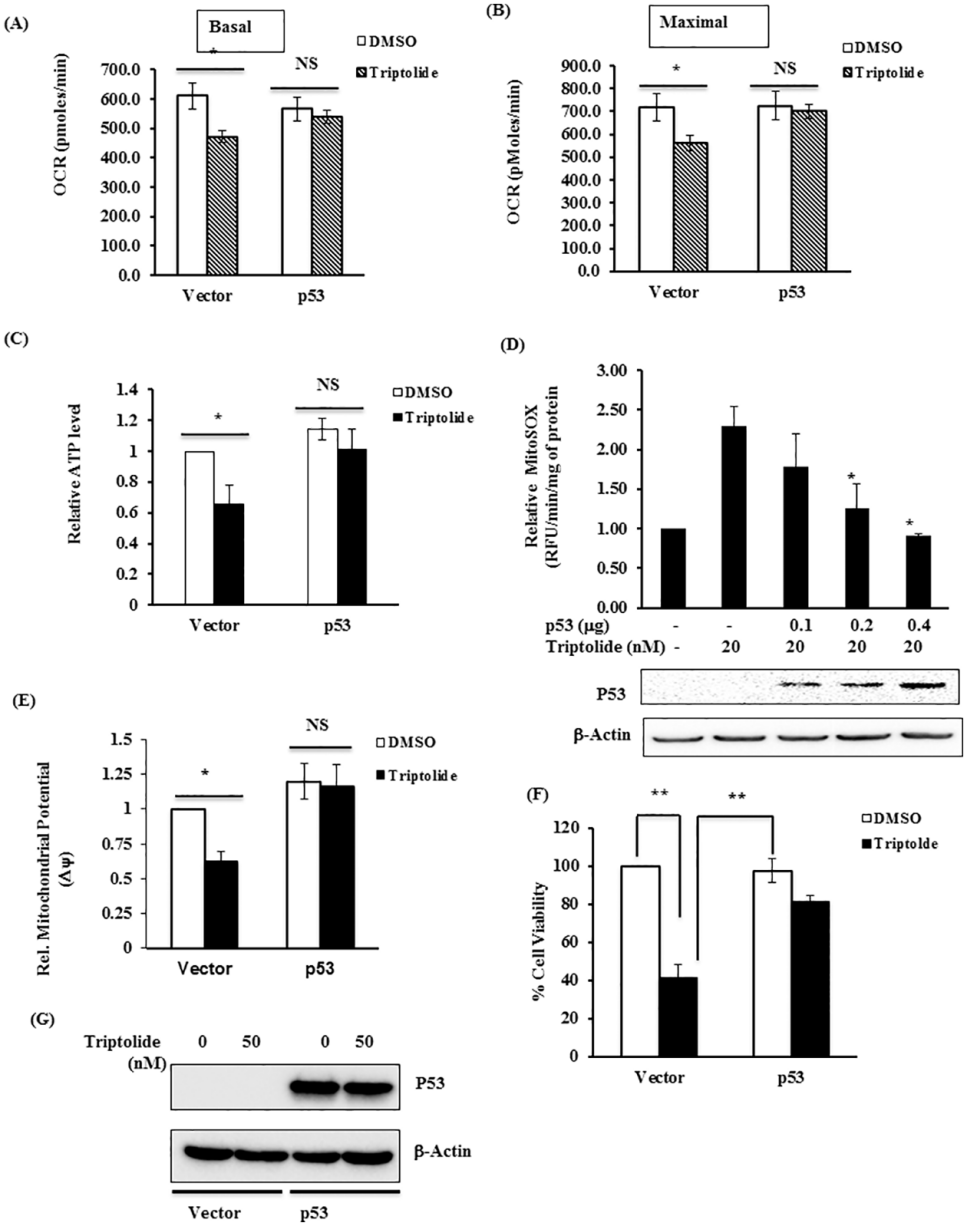
P53 overexpression prevents TL-induced mitochondrial dysfunction in HCT116 p53-/- cells. (A-B) HCT116 p53-/- cells were transiently transfected with control vector or p53 expression plasmid. After 48 hours of transfection, cells were stimulated with vehicle (DMSO) or TL (25 nM for 6 hours) before subjected for OCR analysis using Seahorse Bioscience Extra Cellular Flux analyzer. In similar experimental conditions, p53 and control vector transfected cells stimulated with DMSO or TL were also analyzed for (C) ATP level, (D) mitochondrial ROS production (MitoSOX) and (E) Mitochondrial membrane potential (ΔΨ). The data was presented as fold change over DMSO-treated vector-transfected cells. (F) P53 renders TL-mediated cell death in HCT 53-/- cells. Cell viability was assessed based on nuclear DNA content by CyQount Cell proliferation assay kit. The data was presented as %viable cells over vector-transfected DMSO-treated cells (mean±SD; *p<0.05, **p<0.001; n = 3). (G) Protein lysate from above experiment were subjected for immunoblotting for P53 to assess the P53 overexpression in HCT116 p53 -/- cells.

We next asked the question whether transient reconstitution of p53 in the background of p53 deleted HCT116 cells could restore TL-mediated mitochondrial dysfunction. To achieve this, HCT166 p53-/- cells were transected with p53 expression plasmid followed by treatment with TL and then mitochondrial functions (OCR, ATP, mtROS andΔΨ) along with cell viability were measured. Indeed, p53 in the background of p53-/- cells does improve TL-mediated decreased basal and maximal OCR (Fig 3A and 3B). Concomitant with OCR data, TL-mediated down-regulation of ATP levels (Fig 3C) and membrane potential (Fig 3E) were diminished in HCT116 p53-/- cells with overexpression of p53. Similarly, p53 overexpression decreased mtROS production, induced by TL (Fig 3D). Improved mitochondrial function in HCT116 p53 -/- with presence of exogenous p53 was clearly reflected with decreased cytotoxicity of TL as measured by cell viability (Fig 3F). As shown in Fig 3G, we confirmed the overexpression of P53 by immunoblotting. These observations suggest that p53 can rescue the detrimental effect of TL.

### TL induced oxidative stress in a p53-depleted cells

**Fig 4:**
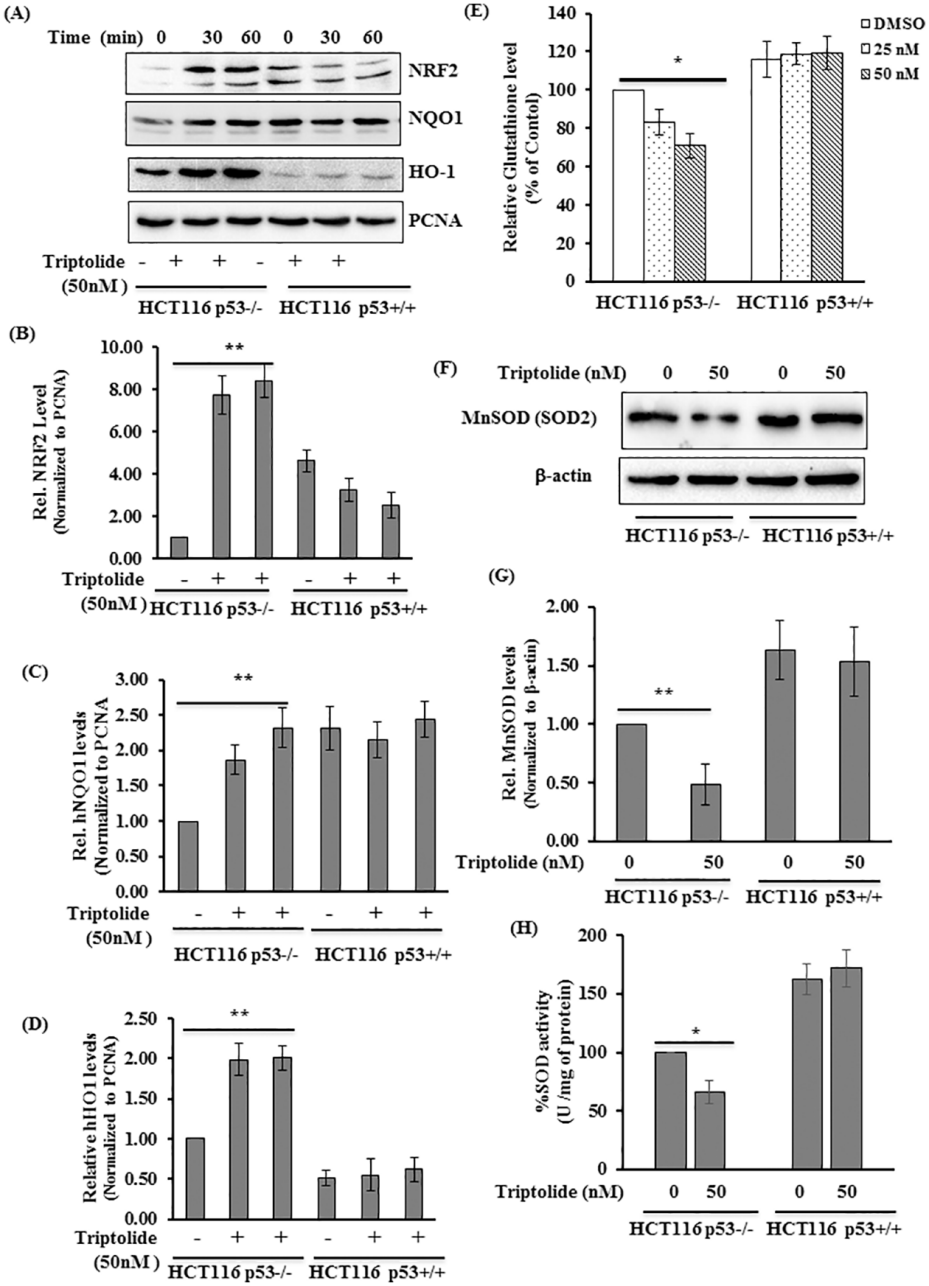
TL induces oxidative stress in p53-Deficient HCT116 cells. (A,B,C) TL treatment led to nuclear accumulation of NRF2 and its target genes (HO1 and NQO1). (A) HCT116 p53+/+ and p53-/- cells were treated with TL (50nM for 30 and 60 minutes). Nuclear extracts were prepared and subjected to immunoblotting with NRF2, NQO1 and HO1. Representative Immunoblots are presented. (B,C,D) Relative protein quantity normalized to PCNA is presented (**p<0.01, n = 3). (E) TL decreases glutathione content. The glutathione levels were measured in cell lysates from TL-induced and control HCT116p53+/+ and p53-/- cells. The levels were normalized with protein content and the data was represented as relative content of %control (*p<0.05). (F-H) TL inhibits SOD2 levels and activity in p53-depleted cells. (F,G) Immunoblotting for Mn-SOD (SOD2) was performed in cell lysates from HCT116 p53+/+ and p53-/- stimulated with DMSO or TL. Relative SOD2 protein levels normalized to beta-actin. (H) SOD activity was measured in cell lysates from F. The data was presented as %activity (mean±SD; *p<0.05; n = 3).

In recent years, several studies have supported that TL-induced cytotoxicity is a result of oxidative stress [46]. The redox-sensitive transcriptional factor, NF-E2 related factor 2 (NRF2) is believed to be a major sensor of oxidative stress. To investigate its role, we next examined NRF2 and its target gene Heme-oxygenase-1(HO1) and NAD (P) H: quinine oxidoreductase-1(NQO1) in the presence of TL. Stimulation of HCT116 p53-/- with TL led to nuclear accumulation of NRF2 within 30 minutes when compared with unstimulated cells (Fig 4A and 4B). Concomitant with NRF2 expression, nuclear hHO1 and hNQO1 were also upregulated in a similar fashion (Fig 4A–4D). Interestingly, in similar experimental conditions, we did not observe any significant difference for nuclear NRF2 or HO1/NQO1 in TL-treated HCT116 p53+/+ cells (Fig 4A and 4D).

Oxidative Stress is a complex and dynamic scenario where the balance between the production of ROS and the presence of antioxidants is disturbed. Among many antioxidant molecules, glutathione is the most abundant antioxidant molecule in the cells [47]. Therefore, we next evaluated the GSH pool of HCT116 cells in presence of TL. As expected, TL considerably reduced total GSH level in HCT116 p53-/- cells when compared with control cells (30–35% reduction) (Fig 4E). However, GSH levels were unchanged in TL-treated p53+/+ cells. Finally, we extended these results by examining the impact of TL on the expression of manganese superoxide dismutase (Mn-SOD/SOD2), a superoxide dismutase localized to the mitochondrion. TL reduced the expression of SOD2 in p53-/- cells without altering the expression in TL-stimulated p53 containing HCT116 cells (Fig 4F and 4G). Consistent with protein level, SOD activity was also decreased in TL-treated HCT116 p53-/- cells with minimal changes in p53+/+ cells. Taken together, these data suggested that presence of p53 dictates oxidative stress caused by TL.

### TL disrupted ETC Complex activities in p53-/- cells

**Fig 5:**
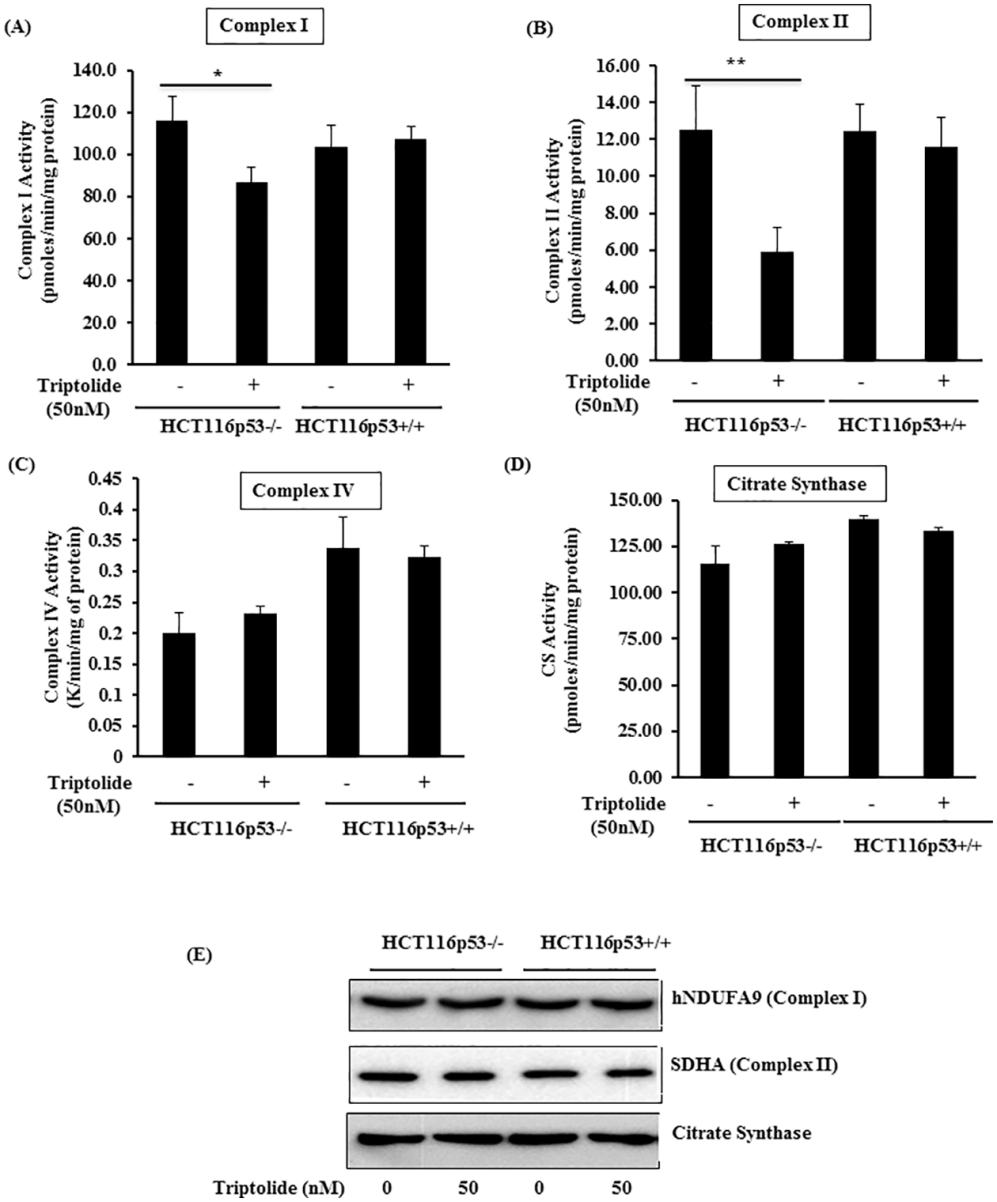
TL stimulation exhibited blunt activities of Complex I and II in p53-deficient HCT116 cells. (A-D) Specific enzymatic activities of ETC complex I, II, IV and of citrate synthase (CS) were measured cells lysates from TL (50 nM) stimulated or control HCT116 P53+/+ and HCT116p53-/- cells. (E, F) Representative Immunoblots for hNDUFA9 (complexes I), hSDHA (Complex II), and citrate synthase are presented (mean±SD; *p<0.05; n = 3).

Armed with the knowledge that TL alters OCR, ATP levels, and mtROS production, we next asked if TL modulates the activity of specific enzymatic complexes of the electron transport chain (ETC). To address this, Complex I, II and IV activities were measured in the mitochondrial fraction of the cells. As compared to control (DMSO) cells, TL treated HCT116 p53-/- cells displayed significantly decreased specific enzymatic activities of complex I and II (Fig 5A and 5B). In contrast, HCT116 p53 +/+ cells revealed no difference in complex I and II activity in the presence of TL (Fig 5A and 5B). Notably, complex IV and citrate synthase activity were unchanged in similar experimental conditions in both the HCT116 cells (p53+/+ and p53-/-) suggesting the specificity of the TL effect (Fig 5C and 5D). Protein expression studies demonstrated no noticeable difference for protein levels of complex I (hNDAUF9) and complex II (SDHA) following stimulation of HCT116 with TL (Fig 5E). These findings support that TL-induced inhibition of complex activities are specific in p53 deficient cells and are not due to changes in protein expression.

### TL promoted hyperacetylation of Complex I and II through mitochondrial Sir3

**Fig 6:**
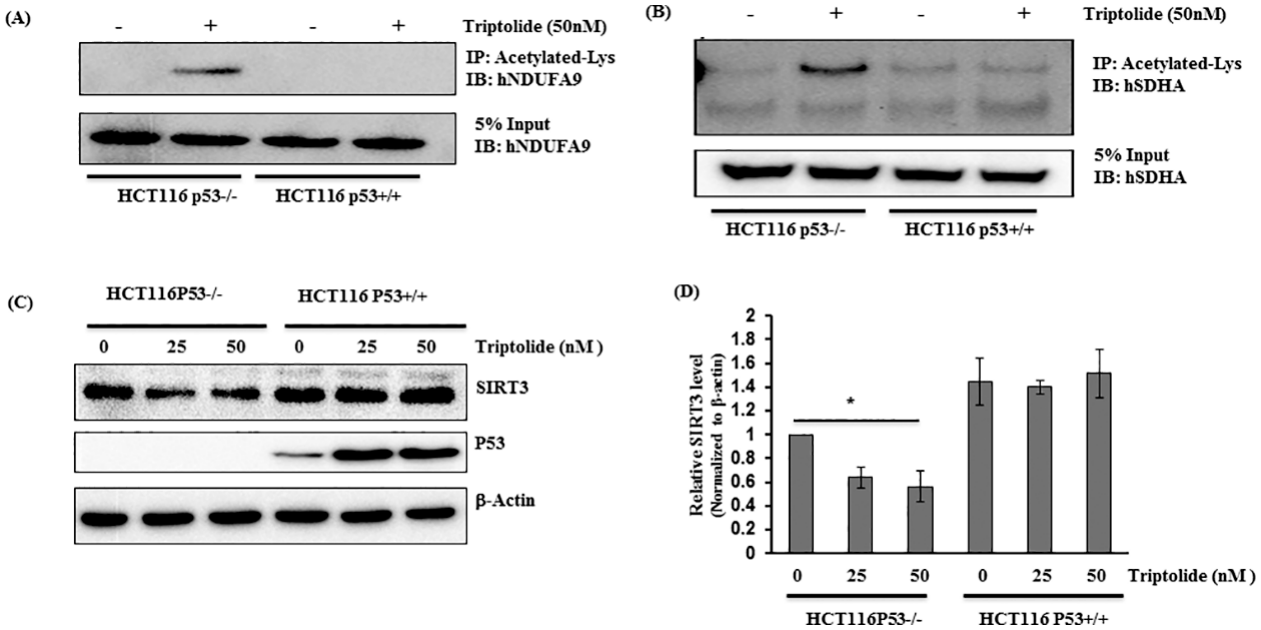
TL induces hyperacetylation of ETC complexes through mitochondrial Sirt3. (A,B) P53 containing and deficient HCT116 cells were treated with TL (50 nM for 6 hours). Immunoprecipitates of anti-acetylayed lysine were immunoblotted with hNDUFA9 (A, top panel) or succinate dehydrogenase (hSDHA) (B, top panel). Whole-cell lysates were immunoblotted with hNDUFA9 (A, lower panel) and SDHA (B, lower Panel) for the input. (C) TL down-regulates SIRT3 expression. Whole-cell lysates from HCT116 cells (p53 +/+ and p53-/-) induced by DMSO or TL were immunoblotted with anti-SIRT3, P53, and beta-actin (top panel). Relative quantity of SIRT3 was computed after normalization with beta-actin (mean±SD; *p<0.05; n = 3).

The enzymatic activities of the complexes of the ETC are regulated through post-translational modifications. For instance, hyperacetylation of complex I and II in mitochondrial diminish their enzymatic activities [32, 33]. Based on this knowledge, we next evaluated whether TL could modulate the acetylation state of ETC complexes to regulate their enzymatic activities and whether p53 plays any role in such an event. To address this, HCT116 p53+/+ and p53-/- cells were stimulated with TL (50nM for 4 hours) and acetylation of NDUFA9 (one of the component complex I) and succinate dehydrogenase (SDHA, Complex II) were assessed. Indeed, we observed increased acetylation of NDUFA9 (Fig 6A) and SDHA in TL treated HCT116 p53-/- (Fig 6B). Intriguingly, the presence of p53 diminishes TL-mediated hyperacetylation of NDUFA9 or SDHA (Fig 6A and 6B). Taken together, these findings demonstrate that TL treatment in p53 deficient cells led to post-translational modification of ETC complexes and diminishing their activities.

Sirtuins (Sirt1-7) are class III histone deacetylases that regulate key cellular function by deactylating various proteins. Among these, Sirt3 is a predominant mitochondrial deacetylase that regulates mitochondrial function through deacetylation of multiple proteins of the electron transport chain (ETC) and dictates the acetylation state of mitochondrial proteins [28]. Therefore, we directly assessed the effect of TL on Sirt3 expression on the background of p53 status. In HCT116, in which p53 was knocked-down, SIRT3 expression was significantly reduced in TL-treated cell lysates when compared with unstimulated cells (40–50% reduction) (Fig 6C and 6D). In similar experimental conditions, no noticeable changes were observed for SIRT3 level in TL-treated HCT116 p53+/+ cells. Interestingly, TL stabilizes P53 expression in HCT116 p53+/+ cells suggesting a potential role of p53 in the TL-mediated response through Sirt3 regulation. Altogether, our results demonstrated that TL mediated mitochondrial dysfunction involves mitochondrial Sirt3.

### P53 regulates steady-state level of Sirt3 through its post-transcriptional modification

**Fig 7:**
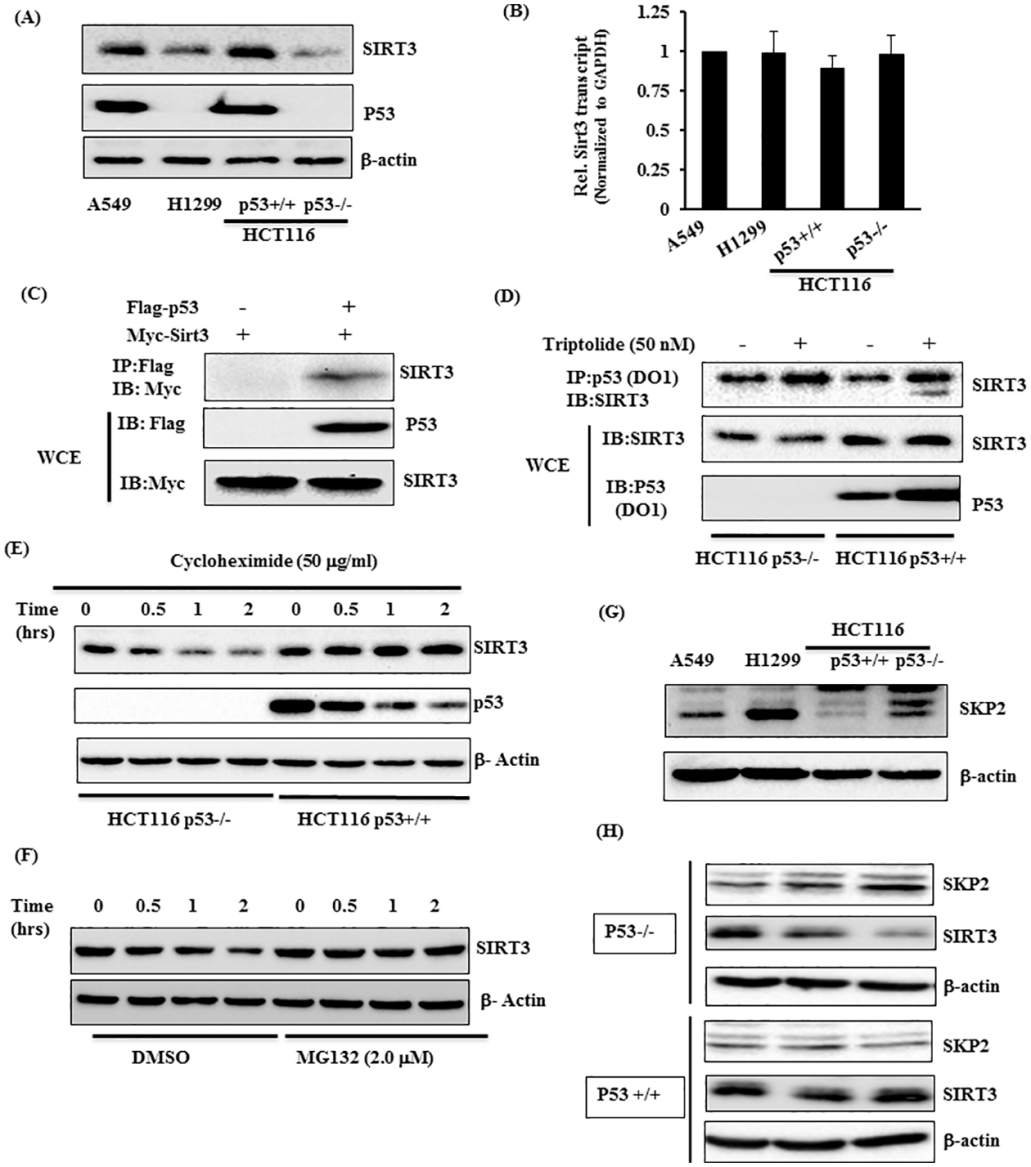
Human P53 associates with M0069tochondrial Sirt3 and modulates its level through Proteasome Pathway. (A, B) p53 regulates Sirt3 level. Sirt3 expression at RNA (A) and protein level (B) were measured in p53 wild type (A549 and HCT116 p53+/+) and p53 deficient cells (H1299 and HCT116 p53-/-). Data are presented relative Sirt3 mRNA in p53 containing cells. (C, D) P53 interacts with SIRT3 in-vivo. HCT116 p53-/- cells were transfected with FLAG-p53 and Myc-Sirt3 alone or in combination. Twenty-four hours post-transfection, cells were harvested in immunoprecipitation assay buffer (IP Buffer). Cell lysates were immunoprecipitated with anti-FLAG followed by immunoblotting with anti-Myc. Five percent of whole-cell extract (WCE) was used as an input and was directly immunoblotted with respective antibodies. (D) Interaction of endogenous P53 and SIRT3. HCT116 p53+/+ cells were stimulated with Triptolide (50 nM) for 6 hours. Cells were harvested in IP buffer and cell lysates were immunopreipiatated with P53 (DO1) antibody followed by immunoblotting with SIRT3 antibody. Five percent WCE was directly immunoblotted with SIRT3 and P53 antibody. (E) SIRT3 turnover in p53+/+ and p53-/- HCT116 cells. Cells were incubated with 50 μg/ml of Cycloheximide for the indicated time. Cell lysates were subjected for immunoblotting with SIRT3 (above panel), p53 (Middle Panel) and β-actin (lower panel). (F,G) SIRT3 degradation is through Proteasome Pathway involving SKP2 E3-ligase. HCT116 p53-/- cells were pretreated with MG132 (2 μM) for 1 hour and then were treated with Cycloheximide (50 μg/ml) for indicated time. SIRT3 and β-actin levels were detected by immunoblotting with respective antibodies. Indicated cell lysates were immunoblotted with anti-SKP2 to examine the expression of SKP2 level (G). (H) TL alters SKP2 levels in p53-/- cells. HCT116 (p53+/+ and p53-/-) cells were stimulated with indicated dose of TL for 6 hours. Cell lsyates were immunoblotted with anti-SKP2, anti-SIRT3 and anti-beta Actin antibodies.

Our observations that TL stabilizes P53 level and fail to alter Sirt3 levels tempted us to investigate the potential role of p53 in Sirt3 regulation. To address this, we first measured SIRT3 protein level in p53 wild type (A549 and HCT116 p53+/+) and p53 deficient cells (H1299, HCT116P53-/-). Cells with functional p53 contained significantly increased SIRT3 level as compared to p53 deficient cells (Fig 7A). Decreased SIRT3 level in p53 depleted cells doesn’t seems like due to transcriptional regulation as we did not observe any changes Sirt3 transcripts between p53+/+ and p53-/- cells (Fig 7B). In an effort to elucidate alternate mechanism for p53-mediated regulation of Sirt3 expression, we then asked if P53 physically associate with SIRT3. To address this, we performed co-immunoprecipitation studies using FLAG-p53 and MYC-Sirt3 in p53-/- HCT116 cells. As noted in Fig 7C, immunoprecipitation with FLAG followed by immunoblotting with Sirt3 showed the association of P53 with SIRT3 (Fig 7C). Consistent with this observation, endogenous p53 also clearly associates with Sirt3 in TL-stimulated HCT116 p53+/+ cells. Given that Sirt3 transcript did not change in p53+/+ and p53-/- cells, decreased Sirt3 level in p53-/- cells may be at post-transcriptional level. To fully understand the dynamics of this, we measured stability of SIRT3 protein in p53+/+ and p53-/- HCT116 cells upon stimulation with Cycloheximide, an inhibitor of protein synthesis. Consistent with decreased SIRT3 levels in p53-/- cells, we noticed significantly reduced stability of SIRT3, exhibiting a half-life of less than an hour. Nonetheless, half-life of SIRT3 in HCT166 p53+/+ cells was more than two hours (Fig 7D). To ascertain if the ubiquitin-proteasome pathway is required for SIRT3 degradation in p53-/- cells, we assess the half-life of SIRT3 in p53-/- HCT116 cells in presence of MG132, an inhibitor of Proteasome- pathway. As shown in Fig 7E, pretreatment of p53-/- with MG132 prevents SIRT3 degradation by extending its half-life. To further strengthen our observation about involvement of proteasome pathway for the increased turnover of SIRT3 in p53 depleted cells, we examined the expression of Skp2 E3 ligase that has previously been shown to affect steady-state level of SIRT3 [48]. Remarkably, cells depleted in p53 (H1299 and HCT116 p53-/-) displayed increased level of SKP2 (Fig 7F) that may be responsible for the increased degradation of SIRT3 in p53 deficient cells. Most importantly, stimulation of HCT116 p53-/- cells with TL increases SKP2 level which coincide with decreased Sirt3 level. In our experimental conditions, not much changes for SKP2 or SIRT3 level in p53+/+ cells following TL treatment. Overall, these finding clearly demonstrate that p53 may regulate SIRT3 protein expression through Proteasome-Pathway and may involve SKP2 E3 ligase.

### SIRT3 attenuated TL-induced mitochondrial dysfunction in p53-deficient cells

**Fig 8:**
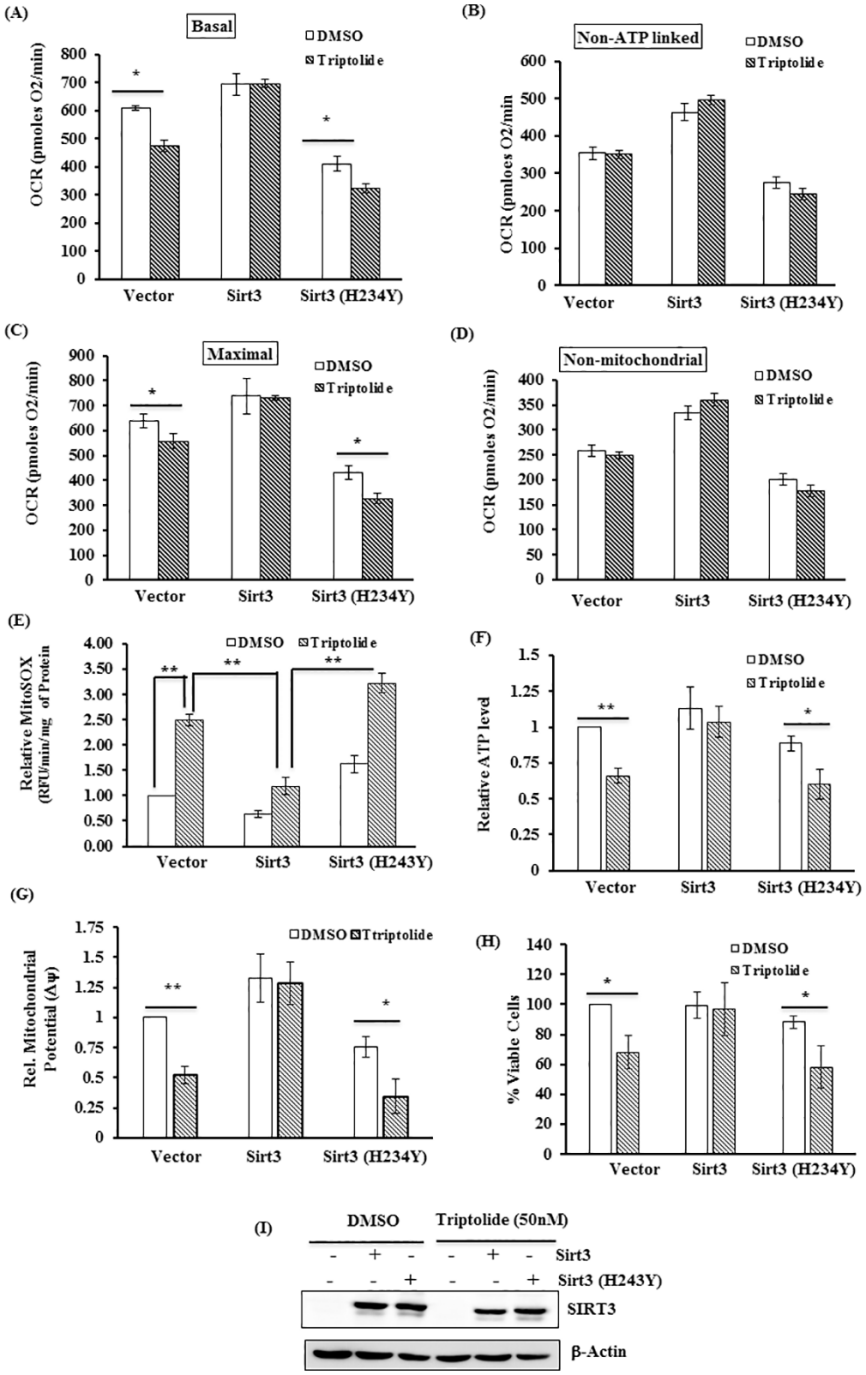
Mitochondrial SIRT3 mitigates TL-induced mitochondrial dysfunction in p53-deficient cells. (A-D) SIRT3 overexpression rescues mitochondrial respiration in TL-stimulated HCT116 p53-/- cells. P53 deficient HCT116 cells were transfected with vector, myc-SIRT3 or myc-SIRT3 (H243Y). 24 hours of post-transfection, cells were stimulated with TL (50 nM) for 6 hours and were subjected for analyzing oxygen consumption rate (OCR) using the Seahorse Bioscience Extra Cellular Flux analyzer. Data were normalized with protein content and presented as basal (A), non-ATP-linked (B), maximal (C) and non-mitochondrial respiration (D) (mean±SEM; *p<0.05; n = 3). (E-G) SIRT3, but not deacetylase deficient mutant of SIRT3, abrogates TL-induced alteration in ROS production, membrane potential and ATP level. HCT116 p53-/- cells were transiently transfected with indicated plasmids. After 24 hours of transfection, cells were stimulated with TL (50 nM) for additional 6 hours. Mitochondrial ROS (mtROS) (E), ATP level (F) and mitochondrial membrane potential (G) were measured. Relative values were presented as fold change over control ((mean±SD; **p<0.001; n = 3). (H) Cells transfected with Myc-SIRT3 or Myc-SIRT3 (H243Y) were stimulated with 50 nM of TL and cell viability was assesses using CyQuont cell proliferation assay kit. The data was presented as % viable cells over vector transfected DMSO stimulated cells. (I) Protein lysates from vector, Myc-SIRT3 or Myc-SIRT3 (H243Y) transfected HCT116 p53-/- cells were immunoblotted with Myc antisera to assess the expression of overexpressed SIRT3 or SIRT3 (H243Y) mutant.

Finally, we determined whether overexpression of SIRT3 in p53 deficient HCT116 rescues mitochondrial dysfunction induced by TL. As a first step, we measured mitochondrial respiration in HCT116 p53-/- transfected with wild-type or deacetlyase-deficient mutant SIRT3 (H243Y) in the presence of TL. Consistent with Fig 2A, TL significantly decreased basal and maximal respiration in vector transfected cells (Fig 8A–8C). Remarkably, overexpression of wild-type SIRT3 completely rescued TL-induced down-regulation of basal and maximal respiration (Fig 8A–8C). Importantly, the SIRT3 mutant (H243Y) was unable to recover triptolide-mediated decreased mitochondrial respiration (Fig 8A–8C). In agreement with previous studies, SIRT3 overexpression increased the overall respiration, whereas SIRT3 (H243Y) mutant appeared to have a dominant negative effect as it reduced overall mitochondrial respiration. These observations encouraged us to examine the other mitochondrial parameters such as mitochondrial ROS production, membrane potential, ATP levels and the cell viability in SIRT3 overexpressing cells. As expected, TL stimulated mtROS production, decreased membrane potential and ATP levels in cells transfected with vector control. Nevertheless, SIRT3, but not SIRT3 (H243Y) mutant, overexpression significantly diminished the effect of TL on ROS production (Fig 8E), ATP levels (Fig 8F) and mitochondrial membrane potential (Fig 8G) and. Nevertheless, overexpression of SIRT3 (H243Y) mutant failed to recuperate any of the mitochondrial function (e.g. OCR, MtROS, membrane potential and ATP levels) in cells stimulated with TL (Fig 8E–8G). Consistent with mitochondrial function, wild type SIRT3, but not the mutant SIRT3, was able to revoke the TL-mediated cell death (Fig 8H). Overexpression of transiently transfected SIRT3 or SIRT3H243Y was confirmed by immunoblotting (Fig 8I). Overall, these findings suggested that activation of mitochondrial SIRT3 could protect the cells against the development of TL-stimulated mitochondrial dysfunction.

**Fig 9:**
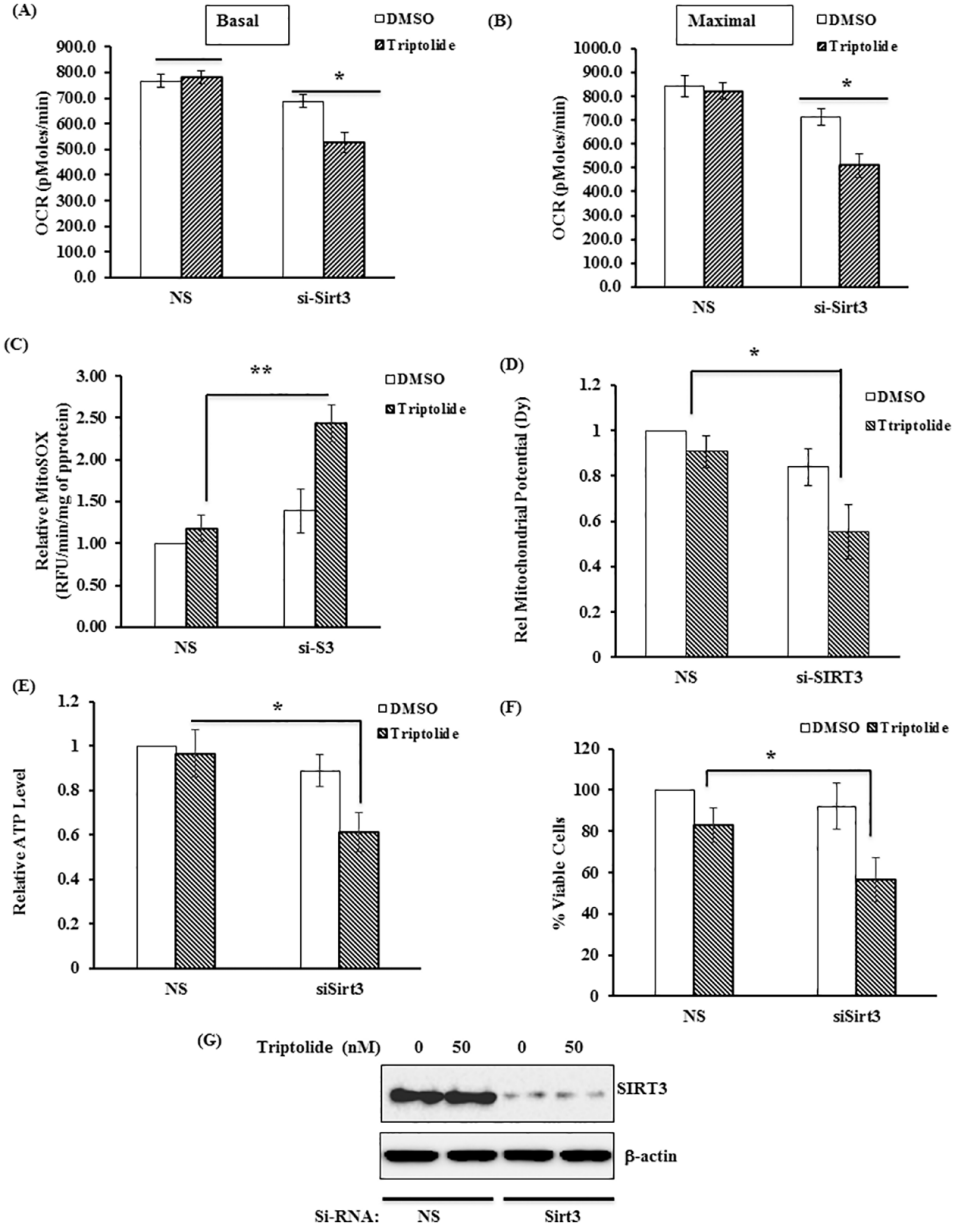
Silencing of Sirt3 sensitizes HCT116 53+/+ cells for TL-mediated mitochondrial dysfunction. (A-B) HCT116 p53+/+ cells were transfected scrambled siRNA (NS) or Sirt3 siRNA. After 48 hours, cells were treated with 50 nM Triptolide tor additional 6 hours before subjected for Seahorse XF analyzer for OCR. Basal (A) and Maximal (B) respiration were compared among the groups. (C-F) Cells from above (A,B) were also analyzed for (C) ATP level, (D) MitoSOX for mtROS, (E) Mitochondrial potential and (F) cell viability. (G) Cells from above were also subjected for cell viability assay. The data were expressed relative over NS-siRNA transfected cell treated with DMSO (mean±SD; *p<0.05, **p<0.001; n = 3)

To further elucidate the mechanism behind the protective effect of Sirt3 for TL-induced effect, we down-regulated Sirt3 in HCT116 p53+/+ cells and asked whether deficiency of Sirt3 in the background of p53 would sensitize the cells for TL mediated effect. As expected, siRNA-mediated down-regulation of Sirt3 in p53 containing HCT116 cells decreases the basal and maximum respiration when stimulated with TL (Fig 9A and 9B). More importantly, we observed alteration of mitochondrial function (decreased mitochondrial potential, lowered ATP and increased mtROS) in Sirt3-downregulated cells (Fig 9C–9E). Remarkably, TL-mediated dysfunctional mitochondria was very comparable with findings in TL-stimulated p53 deficient cells. Finally, these effects are clearly reflected as decreased cell viability in Sirt3 knocked-down cell treated with TL (Fig 9F). Altogether, diminished Sirt3 expression could be responsible for TL-induced mitochondrial dysfunction and increased cytotoxicity in p53 depleted cells.

## Discussion

NSCLC is the most common malignancies world-wide and there is a clear need for effective therapies. We previously demonstrated the *in vitro* and *in vivo* anti-tumor properties of TL suggesting its potential as a promising agent for the treatment of NSCLC. We demonstrated that TL profoundly reduced the expression of anti-apoptotic genes while upregulating pro-apoptotic gene via NF-kB pathway [10]. In those studies, the differential responsiveness of various NSCLC tumors was observed, but the mechanism for this was not delineated. In the present study, we demonstrate that p53 deficiency exacerbates cytotoxic effect of TL (Fig 1 and S1 Fig). Moreover, TL promotes dysfunctional mitochondria in p53-depleted cells through modulation of sirt3 expression.

We extended our previous work that TL promoted apoptosis in various lung epithelial cells by regulating key apoptotic factors. In this study, we examined the significance of p53 for cell in presence of lower doses of TL. P53 plays a key role in cell viability and proliferation. In addition, p53 is well known as a major mediator of various cellular events prompted by stress. Interestingly, p53 deficiency renders cancer cells to enter in the S and M phase instead of cell cycle arrest. In these circumstances, p53 deleted/mutated cells when stimulated with drug exhibit significantly more cells death as compared to their wild-type counter parts due to absent cell cycle arrest coupled with DNA damage that activates mitotic catastrophic events [49]. Lastly, there is an immense relevance of crosstalk between two transcriptional factors, p53 and NF-kB in cancer and other diseases for chemotherapeutic potential. Consistent with prior studies [50], we also demonstrate that p53 depletion resulted reduced nuclear p65 levels with decreased NF-kB transcriptional activity (S4 Fig). Interestingly, depletion of p53 in normal epithelial cells displays some toxicity of TL (data not shown). Nonetheless, TL effect was more robust in p53 deficient transformed cells (e.g. HCT116) implying the selective cytotoxicity of TL in p53 deficient/mutant cancer cells.

The p53 protein has been well recognized for its role in nuclear DNA repair, one of the mechanisms for tumor inhibition. Nevertheless, there is a growing evidence that p53 also modulates mitochondrial function. Under stress, p53 translocate to mitochondria and interacts with mtDNA repair machinery to correct the damage [51]. This event of translocation of P53 to mitochondria is believed to be through mono-ubiquitination of p53 [52]. In addition, loss of p53 is associated with mitochondrial dysfunction with increased ROS production [53]. Such high ROS levels in cancer cells with dysfunctional mitochondria may be advantageous for drug therapy. In agreement with this, we observed TL induces mitochondrial dysfunction demonstrated by decreased OCR, increased mtROS, and decreased ATP in p53 deleted cells (Fig 2). Nonetheless, cells with functional p53 are resistant to such effect of p53 on mitochondrial function. This might due to the fact that TL may increase ROS levels above threshold in already elevated ROS in p53 deficient cells causing cell death, while p53 containing cells with significantly lower ROS level can sustain slight increase in ROS level by TL. As a consequence of increased ROS levels, cells display oxidative stress. In recent years, the role of NRF2 as a master regulator of cellular redox state has been extensively studied. Under condition of cellular stress, NRF2 activation neutralizes the increased ROS level in mitochondria by transcriptionally upregulating antioxidant gene expression [54]. In addition, NRF2 directly regulates the expression of γ-glutamyl cysteine ligase, the rate-limiting enzyme of glutathione synthesis. In cancer, NRF2 can play dual role as tumor suppressor or as an oncogene depending on cell context and the environment [55]. In fact, increased expression of NRF2 has been demonstrated in multiple malignancies including lung [56, 57] and colon [58]. In our study, we demonstrate that p53 deficient HCT116 cells which proliferate better that its isogenic p53 containing cells, have increased level of NRF2. Stimulation of such p53 deficient cells with TL induces oxidative stress, which is reflected by increased nuclear accumulation of NRF2 and increased expression of NRF2 target genes (HO1 and NQO1). In addition, we also provide the evidence that TL decreases the level of GSH and SOD2. Remarkably, this effect of TL was robust in p53 sufficient cells as presence of p53 abolishes TL-induced regulation of NRF2. Our results are consistent with prior studies where TL has been shown to activate NRF2 in various cellular systems [59, 60].

In mitochondria, oxidative phosphorylation produces ATP as a result of transfer of electrons from glycolysis and TCA cycle to oxygen by a series of electron transfer reactions in Complexes I-IV. This process facilitates the reduction of oxygen and the translocation of protons into intermembrane space generating the membrane potential to be used by ATP synthase to generate ATP. Therefore, activities of various complexes of ETC are very crucial for oxygen consumption and generation of ROS. In agreement with blunted oxygen consumption and increased ROS, treatment with TL inhibits Complex I and II activities, but not complex IV, demonstrating the specificity of TL for regulating ETC components. Interestingly, such effect of TL on ETC components was absent in p53 containing cells illustrating the significance of presence or absence of p53.

Post-translational modifications (PTM) of OXPHOS component are the key events by which activities of many complexes are regulated. Reversible acetylation of subunits of complex I and II has been shown to downregulate complex activities. Our results suggest that TL increases acetylation of complex I and II subunits without altering their overall protein expression. Remarkably, such effect of TL on complex I and II modifications was absent in p53 positive cells. These observations are consistent with blunted complex I and II activities in TL-stimulated p53 deficient cells, but not in p53 sufficient cells. Several lines of evidence suggested that acetylation state of mitochondrial proteins are controlled by family of sirtuins and Sirt3 has been shown to be a major deacetylase that regulate mitochondrial function by regulating key metabolic enzymes [28]. Consistent with these findings, we showed that TL blunts the expression of Sirt3 only in p53-depleted cells that directly correlates with increased acetylation of NDUAF9 and succinate dehydrogenase (Fig 6C and 6D). In addition, we also provide the evidence that Sirt3 overexpression can rescue TL-mediated mitochondrial dysfunction. On contrary, knockdown of Sirt3 in p53 containing cells confers sensitivity for TL suggesting that TL may use p53-Sirt3 axis to modulate mitochondrial function. Therefore, it will be worthwhile to fully examine how TL downregulates Sirt3 expression. In this context, studies are underway. A possible explanation for down-regulation of Sirt3 by TL in cells absent in p53 may be due to the fact that TL induces P53 expression, which in turn may increase Sirt3 expression. Supporting this notion, we observed increased turnover of SIRT3 protein in p53 deficient cells through post-transcriptional mechanism. This observation raises a possibility of interaction between P53 and SIRT3. Indeed, we demonstrate that P53 associates with SIRT3 in presence of TL-stimulation implying a novel role of P53 in regulation of SIRT3. Consistent with our results, others have also shown interaction of P53 with SIRT3 in presence of genotoxic stress [61]. Finally, we strengthen our conception by demonstrating that p53 deficient cells contains elevated level of Skp2 E3 ligase which have previously shown to mediate SIRT3 degradation [62]. Given the fact that SIRT3 interacts with Skp2 [62] as well as with p53 [63], it will be worthwhile to investigate the kinetics of this tri-molecular complex if interaction of P53 with SIRT3 could disrupt the SIRT3-Skp2 complex leading to increase level of SIRT3 in p53 containing cells. Lastly, our overexpression studies coupled with knock-down studies with Sirt3 supports an essential role of Sirt3 for TL-induced impairment of mitochondrial function.

In summary, our results demonstrate that mitochondrial Sirt3 plays a fundamental role in regulating TL-stimulated mitochondrial function in p53 dependent manner in NSCLC. P53 deficient cells are more susceptible to TL for dysfunctional mitochondria. These studies provide insight into the mechanistic effects of this drug in NSCLC and will be important pre-clinical investigations for the translation of TL in clinical trials.

## Supporting Information

S1 Fig Representative traces for OCR of TL-treated HCT166 p53-/- (A) and p53+/+ (B) cells. (TIF)

S2 Fig Mito-TEMPO abrogates TL-mediated mtROS accumulation and cell death. **(A, B).** HCT116 p53-/- were incubated with Mito-TEMPO (10mM) and or with TL (25 nM) for 6 hours. Cells were processed for MitoSOX kinetic assay (A) and cell viability (B). The data were presented as relative value over DMSO treated cells. (mean±SD;*p<0.05; ** p<0.001; n = 3).
(TIF)

S3 Fig TL treatment displayed mitochondrial dysfunction in NSCLC. **(A, B).** H1299 cells were incubated DMSO or with TL (25 nM) for 6 hours and were assayed for oxygen consumption rate (OCR) using the Seahorse Bioscience Extra Cellular Flux analyzer. Data were presented as basal, non-ATP-linked, maximal and non-mitochondrial respiration (*p<0.05). (C, D) Cells from (A) were processed for MitoSOX kinetic assay and ATP assay (B). The data were presented as relative value over DMSO treated cells. (mean±SD; *p<0.05; ** p<0.001; n = 3).
(TIF)

S4 Fig P53 modulates NF-kB activity. (A) P53 overexpression displayed increase nuclear p65. HCT116 p53-/- cells were transiently transfected with p53 or control vector plasmids. Nuclear extract were prepared from transfected cells and were immunoblotted with anti-p65, anti-p53 and anti-MSH2. Representative Immunoblot is shown. (B) P53 increases NF-kB transcriptional activity. The activity of 3xNF-kB reporter construct was measured in HCT116 p53-/- cells with and without p53. Normalized (firefly/Renilla) promoter activity is expressed relative to cells with no p53 (**p<0.001, n = 3).
(TIF)
