# Supplementary figures and images for: Minnelide/Triptolide Impairs Mitochondrial Function by Regulating SIRT3 in P53-Dependent Manner in Non-Small Cell Lung Cancer
Source: PLoS One. 2016 Aug 8;11(8):e0160783. doi: 10.1371/journal.pone.0160783 (PMC4976872; doi:10.1371/journal.pone.0160783)

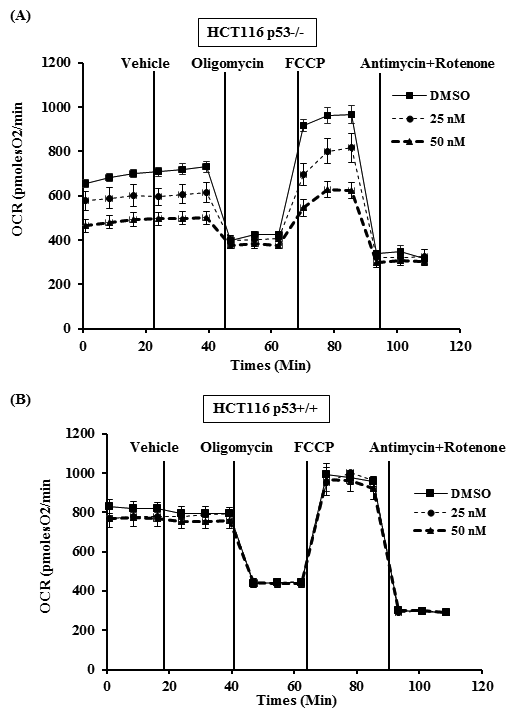

Supplement: S1 Fig — (TIF) [file pone.0160783.s001.tif]

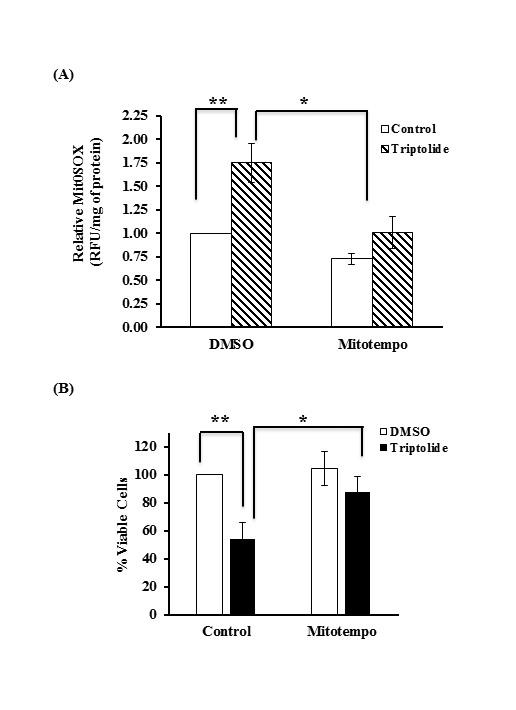

Supplement: S2 Fig — (A, B). HCT116 p53-/- were incubated with Mito-TEMPO (10mM) and or with TL (25 nM) for 6 hours. Cells were processed for MitoSOX kinetic assay (A) and cell viability (B). The data were presented as relative value over DMSO treated cells. (mean±SD;*p<0.05; ** p<0.001; n = 3). (TIF) [file pone.0160783.s002.tif]

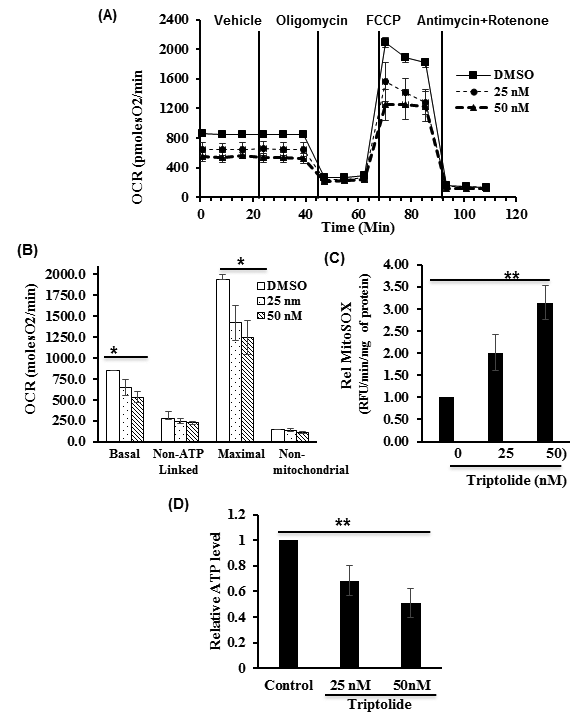

Supplement: S3 Fig — (A, B). H1299 cells were incubated DMSO or with TL (25 nM) for 6 hours and were assayed for oxygen consumption rate (OCR) using the Seahorse Bioscience Extra Cellular Flux analyzer. Data were presented as basal, non-ATP-linked, maximal and non-mitochondrial respiration (*p<0.05). (C, D) Cells from (A) were processed for MitoSOX kinetic assay and ATP assay (B). The data were presented as relative value over DMSO treated cells. (mean±SD; *p<0.05; ** p<0.001; n = 3). (TIF) [file pone.0160783.s003.tif]

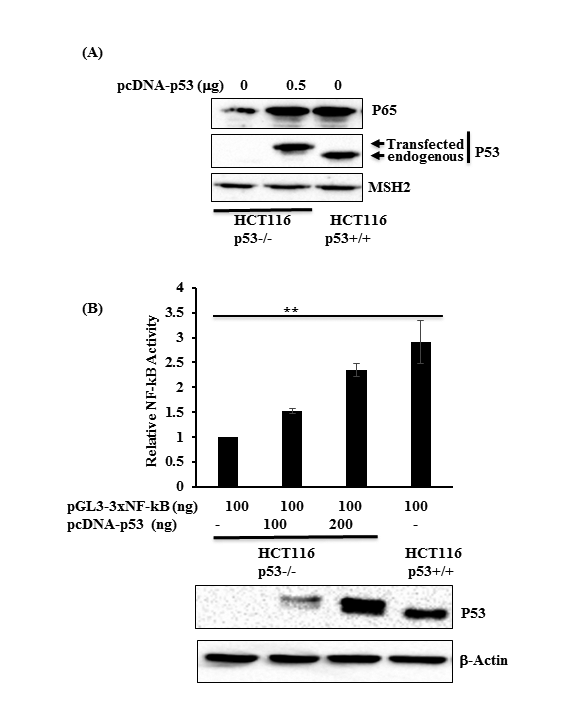

Supplement: S4 Fig — (A) P53 overexpression displayed increase nuclear p65. HCT116 p53-/- cells were transiently transfected with p53 or control vector plasmids. Nuclear extract were prepared from transfected cells and were immunoblotted with anti-p65, anti-p53 and anti-MSH2. Representative Immunoblot is shown. (B) P53 increases NF-kB transcriptional activity. The activity of 3xNF-kB reporter construct was measured in HCT116 p53-/- cells with and without p53. Normalized (firefly/Renilla) promoter activity is expressed relative to cells with no p53 (**p<0.001, n = 3). (TIF) [file pone.0160783.s004.tif]
